# Plasminogen Binding and Activation at the Mesothelial Cell Surface Promotes Invasion through a Collagen Matrix

**DOI:** 10.3390/ijms23115984

**Published:** 2022-05-26

**Authors:** Zachary Ditzig, Caleb M. Wilson, Jesse Salas, Kinta M. Serve

**Affiliations:** Department of Biological Sciences, Idaho State University, 921 S. 8th Ave., Stop 8007, Pocatello, ID 83209, USA; ditzzach@isu.edu (Z.D.); calebwilson1@isu.edu (C.M.W.); jessesalas@isu.edu (J.S.)

**Keywords:** plasminogen, mesothelial cell, plasmin, proteolysis

## Abstract

Plasminogen (Plg) activation to the serine protease plasmin (Pla) plays a key role in regulating wound healing and fibrotic responses, particularly when bound to cell surface receptors. Our previous work suggested that mesothelial cells bind Plg at the cell surface, though no Plg receptors were described for these cells. Since mesothelial cells contribute to injury responses, including cellular differentiation to a mesenchymal-like phenotype and extracellular matrix remodeling, we hypothesized that Plg binding would promote these responses. Here, we confirm that Plg binds to both pleural and peritoneal mesothelial cells via the lysine-binding domain present in Plg, and we demonstrate the presence of three Plg receptors on the mesothelial cell surface: α-Enolase, Annexin A2, and Plg-R_KT_. We further show that bound-Plg is activated to Pla on the cell surface and that activation is blocked by an inhibitor of urokinase plasminogen activator or by the presence of animal-derived FBS. Lastly, we demonstrate that Plg promotes mesothelial cell invasion through a type I collagen matrix but does not promote cellular differentiation or proliferation. These data demonstrate for the first time that mesothelial cells bind and activate Plg at the cell surface and that active Pla is involved in mesothelial cell invasion without cell differentiation.

## 1. Introduction

The plasminogen activation system (PAS) extensively regulates physiologic tissue remodeling during wound healing, coagulation, and fibrinolysis and is also involved in the initiation and resolution of inflammation [1,2]. However, upon chronic inflammation or PAS dysregulation, extracellular matrix (ECM) homeostasis is disrupted, resulting in excess collagen and fibrin accumulation. Pleural fibrosis etiologies include chronic inflammation, infection, surgery, emphysema, and exposure to silicate minerals, including silica dusts and amphibole asbestos fibers [3]. Amphibole exposures in particular are associated with lamellar pleural thickening, a severe, progressive form of pleural fibrosis, though disease pathogenesis is not fully understood [4,5].

Pleural mesothelial cells contribute to local inflammation and tissue injury responses through production of inflammatory cytokines, ECM proteins, and PAS-activating and inhibiting molecules. Recent evidence also indicates that mesothelial cells may contribute by directly binding plasminogen (Plg) proteins [6]. Our previous work identified autoantibodies against a mesothelial cell-bound antigen in people and mice exposed to amphibole asbestiform fibers and demonstrated a significant and positive correlation between autoantibody presence and pleural disease [4,7]. Follow-up studies established Plg as the autoantibody target [6], suggesting that mesothelial cells directly bind Plg at their surface. However, no mechanism of binding has been described, and it is unclear if Plg is activated to plasmin (Pla) on the mesothelial cell surface. It is also unclear what effect active Pla may have on mesothelial cells, though other groups have suggested that Pla induces mesothelial–mesenchymal transition (MMT) [8]. Pla also plays a critical role in cell migration, pericellular proteolysis, and proliferation [1,9,10,11]. Therefore, we hypothesized that Plg binding and activation on the mesothelial cell surface would induce MMT, proliferation, and cell migration.

Plg binding at the cell surface is mediated by plasminogen receptors (Plg-Rs), which are widely distributed across various cell types, including fibroblasts, macrophages, endothelial cells, and carcinoma cells [12,13]. To protect from premature activation, circulating Plg assumes a compact conformation (Glu-Plg). Following binding to a Plg-R, Glu-Plg is cleaved at Lys62, Arg68, or Lys77 [14], resulting in Lys-Plg, which is readily activated to the serine protease Pla by urokinase or tissue-type plasminogen activator (uPa and tPA, respectively) [15,16]. While mesothelial cells are widely reported to produce uPA, uPA receptor, and plasminogen activator inhibitors, mesothelial expression of PlgRs aside from Annexin A2 has not been described [3,17,18]. Due to the ubiquitous expression of many PlgRs, including α-Enolase (ENO-1), Annexin A2 (ANXA2), Histone H2B protein, and Cytokeratin 8 (CK8), we hypothesized that mesothelial cells bind Plg using at least one of these receptors. We also expected that a newly identified receptor, Plg-R_KT_ [19], may contribute to Plg binding. Lastly, we anticipated that PlgR expression would be similar between pleural and peritoneal mesothelial cells as they are derived from the same cellular precursor.

Here, we present evidence that Plg binds similarly to cultured pleural and peritoneal human mesothelial cells and that both cell lines express ENO-1, AnxA2, and Plg-R_KT_. To our knowledge, this is the only evidence of Plg-R_KT_ expression in mesothelial cells. We also demonstrate that Plg activation on the mesothelial cell surface is blocked by uPA inhibitor and FBS presence in cell culture medium. Lastly, we show that Plg induces mesothelial cell invasion through a collagen matrix but does not affect cell proliferation, differentiation, or migration in the absence of collagen. Together, these data suggest a novel role for mesothelial cells in Plg binding and activation and Plg involvement in mesothelial movement through a type I collagen gel.

## 2. Results

### 2.1. Plg Binds to Pleural and Peritoneal Mesothelial Cells

**Figure 1:**
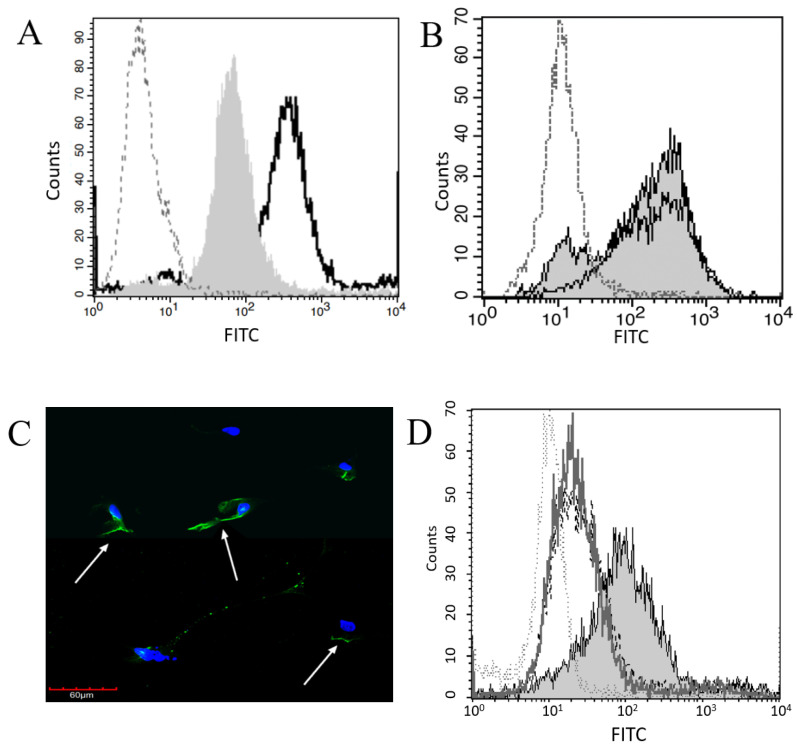
Plg Binds at the Surface of Mesothelial and Macrophage Cells. (**A**) Plg binding to MeT5A cells (grey shaded histogram) was compared to macrophages (–––, unfilled histogram) and isotype control (····). (**B**) Plg binding to MeT5A (grey shaded histogram) and primary peritoneal mesothelial cells (–––, unfilled histogram) was compared to isotype control (····). The Plg binding on all mesothelial cells was significantly higher than isotype control. (**C**) Binding of Plg to MeT5A cells was assessed by confocal fluorescent microscopy. DAPI was used to identify nuclei, and FITC-conjugated anti-Plg antibody used to visualize Plg. Cell surface Plg binding is indicated by white arrows. (**D**) Plg binding was significantly different between MeT5A cells that were serum-starved (grey shaded histogram) grown in 5% FBS (–––, unfilled histogram) or 10% FBS (- - -), and isotype control (····) *p* = 0.05 by one-way ANOVA, *n* = 4.

Plg binding to pleural mesothelial cells (Met5A) and monocyte-derived macrophages (THP-1, used as a positive control) was assessed using flow cytometry. Pleural mesothelial cells showed significantly higher Plg binding than did the isotype control (*p* < 0.01, Figure 1A) but monocyte-derived macrophage cells demonstrated a significant increase in Plg binding compared to mesothelial cells (*p* = 0.03). To ensure that Plg binding is not unique to pleural cells, primary human peritoneal mesothelial cells were examined by flow cytometry and showed significantly higher Plg binding compared to isotype control (*p* = 0.03). Plg binding was not significantly different between peritoneal and pleural mesothelial cells (Figure 1B). Plg binding by pleural mesothelial cells was further confirmed using confocal fluorescence microscopy (Figure 1C). Notably, Plg tended to bind one side of the Met5A cells and did not bind evenly around the entirety of the cell surface, possibly suggesting polarity in these cells. Additionally, flow cytometric assays demonstrated an increase in Plg binding when Met5A cells were serum-starved overnight compared to cells incubated in 5% FBS (Figure 1D). Therefore, all assays were performed on serum-starved cells.

### 2.2. Plg Activation Is Blocked by uPA Inhibitor

**Figure 2:**
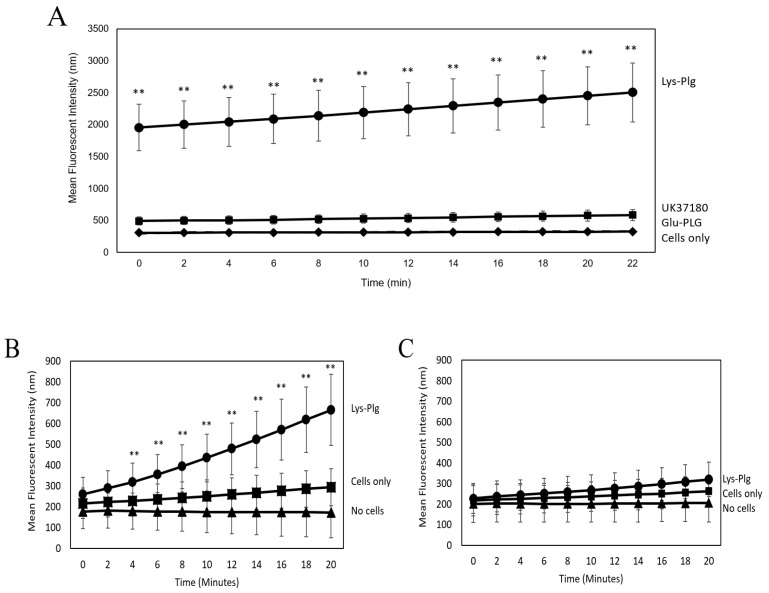
Plg is Activated to Pla Upon Binding to MeT5A Cells. Pla activity assays were used to assess Plg activation to Pla by MeT5A cells. (**A**) Cells were incubated for 2 h with Lys-Plg (circle), Glu-Plg (diamond), or Lys-Plg + uPA inhibitor UK371804 (square), and then the Pla substrate was added. Pla activity was measured in 2 min increments for 21 min following substrate addition. Lys-Plg showed significantly higher Pla activity compared to Glu-Plg or cell-only control (x). UK37180 significantly decreased Pla activity. To confirm Plg binding/activation at the cell surface, Pla activity was also assessed on cells (**B**) or in supernatants (**C**). Following a 1 h incubation with Lys-Plg (circle), cell-surface activity of Pla was significantly higher compared to untreated cells (square) or no cell control (triangle). In contrast, when Pla activity was measured in supernatants alone, no significant activity was noted in any treatment or control group. Each sample was run in triplicate, and experiments repeated at least twice with representative plots shown and data presented as mean ± SD, with ** *p* ≤ 0.001 compared to the untreated control.

The ability of pleural mesothelial cells to activate Glu-Plg and Lys-Plg following binding was determined using a Pla activity assay. A significantly increased amount of Pla activity was measured in cells incubated with Lys-Plg (*p* = 0.001) compared to untreated cells (Figure 2A). As expected, Glu-Plg had significantly lower Pla activity than Lys-Plg at a 2 h time point and looked no different than the cells-only control. This data mirrors our preliminary studies that examined Pla activity following addition of human serum to cells instead of commercial Plg. In these studies, significant Pla activity was seen following a 5 or 12 h cell incubation with serum, but no Pla activity was observed at 15 min or 1 h (data not shown, *n* = 6, *p* < 0.01). Since Glu-Plg is the predominant Plg form in serum, these data confirm what was measured using purified commercial Glu-Plg (Figure 2A).

Since cellular Pla activity is reportedly carried out mainly by uPA [16], and uPA is produced by mesothelial cells [17], we examined Lys-Plg activation in the presence of the uPA inhibitor UK37180 (Figure 2A). Addition of 10 µM inhibitor significantly reduced Pla activity compared to cells incubated with Lys-Plg alone (*p* < 0.01), but activity was still significantly higher than that in untreated cells (*p* = 0.008).

Follow-up experiments were performed to assess purified Lys-Plg activity on serum-starved mesothelial cells. Since Lys-Plg is activated more quickly than Glu-Plg normally found in serum [20], we tested Pla activity at earlier time points than the studies described above. It was found that Pla activity was significantly increased after a 1 h incubation with Lys-Plg compared to untreated cells (*p* < 0.001, Figure 2B). Activity was also examined in cell supernatants alone and revealed no significant differences between supernatants from Lys-Plg treated cells, untreated (control) cells, and background (Figure 2C). Together, these data suggest that Pla activity is dependent on direct Plg–cell interactions, that Pla is active within minutes of Lys-Plg addition, and Plg activity is detectable for at least 12 h following addition to mesothelial cells. Therefore, purified Lys-Plg was used for all subsequent experiments.

### 2.3. Plg Binding and Activity Are Reduced by EACA or FBS

**Figure 3:**
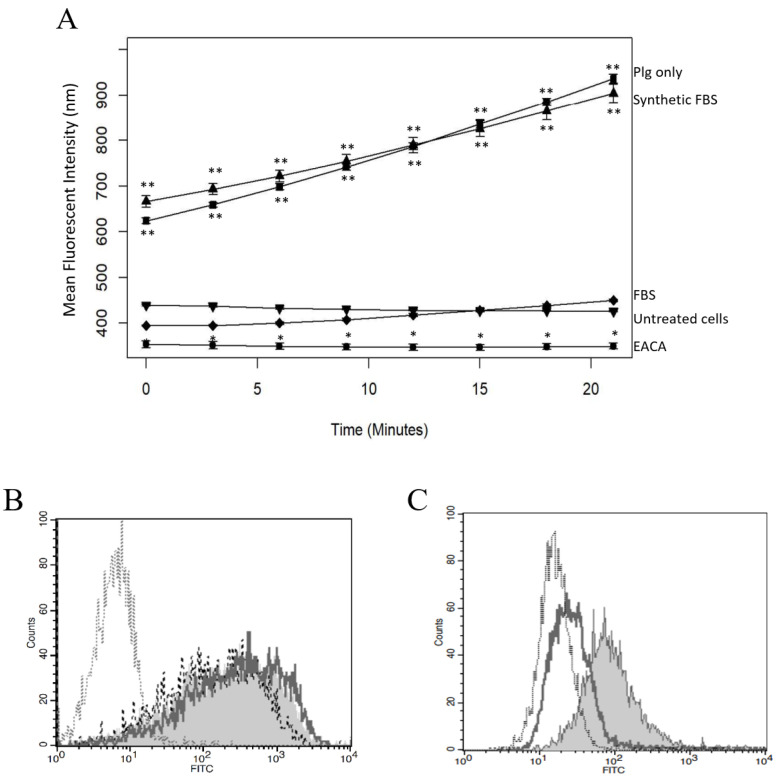
EACA and FBS Inhibit Lys-Plg Binding and Activation on MeT5A Cells. (**A**) Pla activity was significantly decreased when Plg was incubated with 1.6 mM M EACA (circle) compared to Plg only (square) and was similar for the control untreated cells (inverted triangle). Addition of animal-derived FBS (diamond) also significantly lowered Pla activity to negative control levels while addition of synthetic FBS (triangle) had no effect on Pla activity compared to Plg only. Plots are representative of at least three independent trials, *n* = 3 each, with error bars representing SD. Significance is * *p* ≤ 0.01, ** *p* ≤ 0.001, as compared to control. (**B**) Plg binding was not decreased upon addition of animal-derived FBS (- - -) or synthetic FBS (**^_____^**) compared to Plg without FBS (grey shaded histogram), though binding was always higher than isotype control (.....). (**C**) Plg binding was decreased upon addition of 1.6 mM M EACA (**^_____^**) compared to untreated Plg (grey shaded histogram). Plg treatment with EACA reduced binding to levels comparable to the isotype control (.....).

Since many PlgRs are broadly categorized by their ability to bind Plg using a lysine-binding domain (LBD), we examined the effect of the LBD on Plg–mesothelial cell interaction. The addition of a known LBD inhibitor, EACA (1.6 mM, final concentration), significantly decreased Pla activity below levels detected in untreated mesothelial cells (Figure 3A, *p* = 0.01). We also evaluated the effect of FBS on Plg activity. When FBS was added to serum-starved cells just prior to Plg addition, a significant reduction in Pla activity was noted (Figure 3A, *p* < 0.01). Interestingly, addition of synthetic FBS had no effect on Pla activity, suggesting that a component of animal FBS does indeed interfere with Plg activation on the cell surface (Figure 3A). We used flow cytometry to determine if FBS interferes with Plg binding and detected no difference in Plg when FBS was added just prior to Plg (Figure 3B). Addition of Fast-Gro synthetic FBS also had no effect on Plg binding. We further confirmed that EACA does reduce Plg binding compared to isotype control levels (Figure 3C). Together, these data indicate that animal-derived FBS reduces Plg conversion to Pla but does not affect Plg binding to cells, while EACA prevents Plg binding, thus resulting in decreased Pla activity.

### 2.4. Three PlgRs Were Detected on the MeT5A Cell Surface

**Figure 4:**
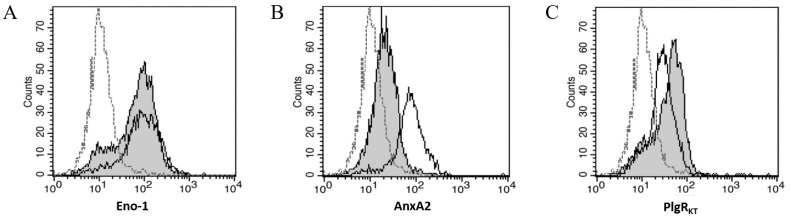
Three Plg-Rs are Detected on Mesothelial Cells; Plg-R_KT_ Contributes to Plg Binding. Representative flow cytometric analyses showed significant expression of (**A**) ENO-1, (**B**) AnxA2, and (**C**) Plg-R_KT_ on MeT5A (–––) and peritoneal mesothelial cell surfaces (grey shaded histogram). In all histograms, isotype control shows little binding (- - -).

Since Plg binding to mesothelial cells relies on the LBD, we used flow cytometry to assess mesothelial cell expression of five widely expressed, LBD-containing Plg-Rs: alpha-enolase (ENO-1), Annexin A2 (AnxA2), Histone H2B protein, Cytokeratin 8 (CK8), and Plg-R_KT_. Three Plg-Rs (ENO-1, AnxA2, and Plg-R_KT_) were detected in both pleural and peritoneal mesothelial cells (Figure 4A–C) and were present at levels significantly higher than isotype control (*p* < 0.01 by one-way ANOVA, *n* = 5); CK-8 and H2B were not detected above isotype control (data not shown).

### 2.5. Plg Does Not Affect MeT5A Proliferation or Differentiation

**Figure 5:**
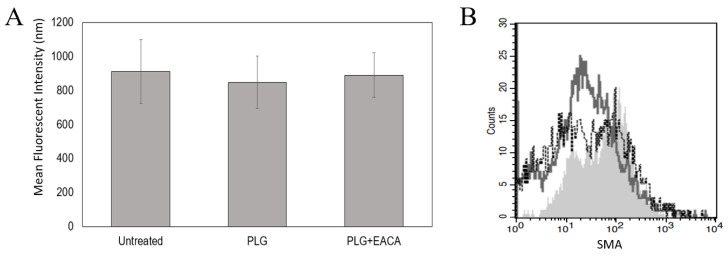
Plg Does Not Affect MeT5A Proliferation or Differentiation. No effect of Lys-Plg on MeT5A proliferation (**A**) and differentiation (**B**) were noted at 48 h post-exposure compared to untreated control cells. (**A**) Proliferation was assessed using CyQuant assays and differentiation measured as increased expression of mesenchymal proteins SMA and vimentin via flow cytometric analyses. (**B**) A representative histogram shows increases in SMA following cell exposure to TGF-beta as a positive control (- - -, unfilled histogram) compared to untreated cells (–––, unfilled histogram). However, no significant effect on SMA was noted following cell incubation with Lys-Plg (grey shaded histogram), *n* = 4.

To examine proliferation, a CyQuant Proliferation Assay (Thermo Fisher) was performed on MeT5A cells incubated with Plg or Plg + EACA for 48 h. Fluorescent intensity was measured as an indicator of DNA content and compared to untreated cells. Overall, no differences were noted between control and treated cells (*n* = 4, *p* = 0.12), suggesting that Plg does not affect MeT5A proliferation (Figure 5A). Since lack of FBS can arrest cell division, we repeated the assay with cells grown in synthetic FBS as opposed to serum-starved. Again, no effect of Plg on cell proliferation was detected (data not shown).

To examine mesothelial cell differentiation, we used flow cytometry to examine changes in smooth muscle alpha-actin (SMA) and vimentin, intracellular proteins increased upon MMT. Cells were incubated with Plg for 48 or 96 h prior to staining as MMT has been noted at both these time points. Flow cytometric analyses revealed increases in SMA and vimentin when cells were incubated with the positive control TGF-β (2 ng), *p* = 0.01 and 0.005, respectively. However, no significant changes in these proteins were noted in cells incubated with Plg compared to untreated cells (Figure 5B). Together these data suggest that Plg does not promote proliferation or MMT in cultured MeT5A cells.

### 2.6. Plg Increases MeT5A Chemotaxis through a Collagen Matrix

**Figure 6:**
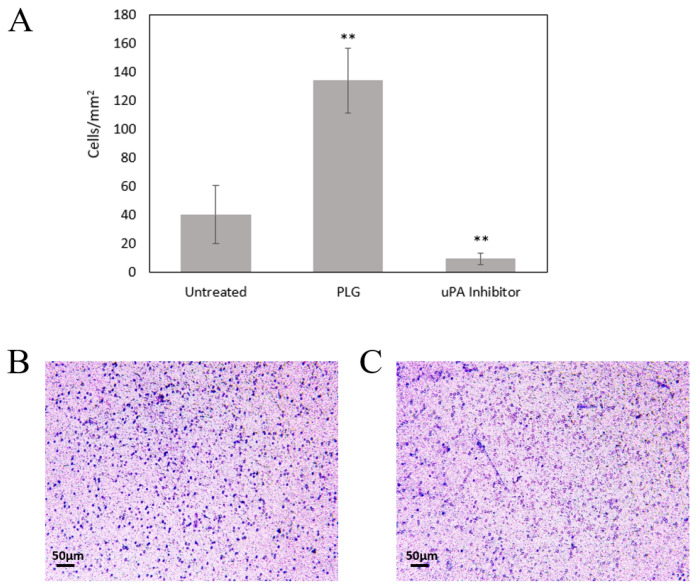
Plg Enhances MeT5A Invasion through a Collagen Matrix. (**A**) Lys-Plg increased Met5A invasion through a 2.65 mg/mL collagen matrix compared to untreated cells (*n* = 3). Addition of uPA inhibitor significantly reduced migration (*n* = 2), ** *p* < 0.001 by one-way ANOVA, mean ± SD. Representative images show cell movement through a collagen-coated Transwell membrane in the presence (**B**) or absence (**C**) of Plg. Cells attached to the lower membrane were fixed and visualized with crystal violet 24 h after seeding in the upper insert.

We utilized Transwell inserts to examine the chemotactic activity of MeT5A cells exposed to Plg. When cells were seeded in uncoated inserts containing 5 µm pores, we detected significant cell migration into the lower chambers, suggesting that mesothelial cells display strong chemotactic activity toward FBS. Additionally, we noted no difference in migration by cells treated with Plg (453 ± 18 cells/mm^2^) compared to untreated cells (455 ± 23 cells/mm^2^). When cells were seeded on top of a type I collagen gel (1.7 mg/mL), we noted a reduction in cell migration, suggesting mesothelial cells do not move as readily through a collagen matrix (352 ± 19 cells/mm^2^). When cells were treated with Plg, significantly (*p* < 0.001) more cells moved through the collagen gel (396 ± 17 cells/mm^2^) compared to the untreated cells. We then tested cell migration using a thicker collagen gel (2.65 mg/mL), which resulted in significantly lower overall cell movement into the lower chambers. However, when cells were treated with Plg, a significantly higher (*p* < 0.001) number of cells migrated into the lower chambers compared to untreated cells (Figure 6). Addition of uPA inhibitor significantly reduced cell migration compared to Plg or untreated cells (*p* < 0.001 by one-way ANOVA). These data suggest that the collagen matrix reduces cellular movement in the absence of the fibrinolytic protein Plg and that Plg promotes mesothelial cell invasion through a collagen barrier.

## 3. Discussion

While mesothelial cells are reported to express various Plg activators and inhibitors, direct Plg binding to mesothelial cells via PlgRs has not been reported in the literature [3,8,17,21]. Using flow cytometry, we confirmed that both pleural and peritoneal mesothelial cells bind Plg, though binding was not as high as measured in monocyte-derived macrophage cells. Importantly, treatment with EACA reduced Plg binding below control levels, indicating that the LBD is critical for Plg binding to mesothelial cells. Therefore, we assessed the mesothelial expression of PlgRs with known LBDs. Using flow cytometry, we confirmed mesothelial expression of three PlgRs, including ENO-1, AnxA2, and PlgR_KT_. These receptors are reported on a number of cell types, but only AnxA2 has previously been identified on mesothelial cells [13,18,22]. Since others have demonstrated the importance of the p11 subunit to AnxA2 Plg binding and activation [23], we included anti-p11 antibodies in some experiments but detected no difference in Plg binding compared to use of anti-AnxA2 antibody alone (data not shown). We used confocal fluorescent microscopy to confirm cell surface binding of Plg and noted that binding fluorescence was not equally distributed around the surface. Since mesothelial cells reportedly have apical–basal polarity and can develop forward–back polarity in some instances [20], it is possible that one surface expresses comparatively more Plg receptors [22]. It is also possible that binding along a single surface could facilitate Plg-mediated activities, such as cell migration and extravasation, as reported in macrophages [2,14]. However, confirmation of the receptor distribution was beyond the scope of this study. Additionally, not all mesothelial cells bound Plg, as determined by both microscopy and flow cytometry, suggesting subsets of mesothelial cells express varying levels of Plg-Rs. Others have reported mesothelial subsets involved in different physiologic responses such as wound healing [3] and have shown apical localization of AnxA2 in mesothelial cells in rat peritoneal tissue [22]. However, additional research is needed to evaluate mesothelial cell population heterogeneity and responses to Plg binding both in vitro and in vivo. 

Pla activity assays were used to assess conversion of Plg to Pla following incubation with mesothelial cells. Unsurprisingly, we noted that, after a 2 h incubation, Lys-Plg was converted to Pla more efficiently than Glu-Plg, likely due to Glu-Plg conformation protecting it from Plg activators [24], thus making activation into Pla slower. Since others reported Plg activation to Pla as early as 15 min [25], we examined the activity of purified Lys-Plg on mesothelial cells following 1 h incubation and noted significant increases in Pla activity. To confirm that Plg/Pla activity was due to cell surface binding, cells and supernatant were assessed separately. Results revealed that only cells showed increased Pla activity, while no activity was noted in supernatants, indicating that Plg/Pla are bound and active at the mesothelial cell surface and not released into the extracellular milieu following Plg activation. Use of the uPA-specific inhibitor (UK37180) also demonstrated that uPA is a major contributor to Lys-Plg activation, a finding corroborated by other research showing significant uPA expression by mesothelial cells [20]. However, these data do not preclude additional Plg activation mechanisms, either by tPA or Pla autocatalysis. While tPA is reportedly produced by mesothelial cells [26,27], further studies are needed to determine if these molecules contribute to overall Plg activation at the mesothelial cell surface, particularly in vivo.

Interestingly, the presence of FBS in the cell culture media reduced activity of bound Plg without blocking its binding. We further demonstrated that synthetic FBS does not affect Plg activity, indicating that the components of animal-derived FBS limit Plg conversion. Bovine serum contains several known serine protease inhibitors (serpins), such as α2-antiplasmin homolog [28,29], which may explain the reduction in Plg/Pla activity when cells were exposed to FBS.

Since PlgRs have not previously been reported on mesothelial cell surfaces, the contribution of Plg binding to physiologic responses is unknown, though the three PlgRs detected are commonly reported on other cell types and contribute to activities such as monocyte migration, macrophage inflammatory responses, and wound healing [2,30,31,32]. Thus, we examined the effect of Plg binding and activation on mesothelial cell proliferation and differentiation to a mesenchymal phenotype. We detected no significant effect of Plg on cell proliferation or differentiation. While exposure to Plg did slightly increase expression of both SMA and vimentin, proteins associated with mesothelial–mesenchymal transition, this change was not statistically significant. Similar results were previously reported by Owens et al., who also showed non-significant increases in SMA and vimentin following mesothelial cell treatment with active Pla [8].

Lastly, we examined the effect of Plg on mesothelial cell migration and collagen matrix invasion using Transwell chambers. No differences were noted in cell migration +/− Plg in the absence of extracellular matrix proteins. The large proportion of migrating cells noted in each group demonstrates that MeT5A cells display strong migratory capacity in the absence of any barrier. However, since mesothelial cells attach to basement membrane proteins in vivo, and Plg/Pla are fibrinolytic, we decided to examine the effect of Plg on migration in the presence of the extracellular matrix protein collagen I. Our initial studies utilized collagen at a final concentration of 1.7 mg/mL. Cells demonstrated a significantly greater invasive propensity through a collagen gel when Plg was present compared to untreated cells. However, at this concentration, collagen protein polymerization results in pores approximately ~5 µm in diameter, which means the cells may have simply moved through the pores and proteolysis was not necessary for movement [33]. Therefore, we repeated the experiments using a final collagen concentration of 2.65 mg/mL to determine if Plg proteolytic activity may be affecting cell migration [33]. When higher collagen concentrations were used, overall cell invasion was reduced but remained significantly higher in cells treated with Plg. The effect of Plg activity on cell movement was further confirmed with the addition of uPA inhibitor UK371804 to the Plg-treated cells. The presence of the inhibitor reduced cell migration to below the untreated cell level, demonstrating that activated Plg contributed to the observed cell movement. Since MeT5A cells are noncancerous, we suspect this invasion demonstrates the ability of Plg-bound cells to remodel ECM proteins, specifically type I collagen, which could contribute to ECM reorganization during physiologic responses such as wound healing. Mesothelial cells have been noted as key players in wound healing responses, including ECM deposition and remodeling [3,34], while Plg/Pla are known regulators of ECM degradation and recycling, tissue remodeling, and angiogenesis during wound healing [1,2]. Thus, activation of Plg on the mesothelial cell surface may contribute to collagen degradation and reorganization, allowing for mesothelial cell movement and, potentially, wound healing or tissue remodeling responses. However, further studies are needed to confirm mesothelial–Plg interaction in physiologic responses and to determine the mechanism by which Plg binding leads to mesothelial cell invasion.

Overall, data presented here demonstrate for the first time that mesothelial cells express three Plg-Rs on their surface and that the LBD is critical for Plg binding and activation to Pla. There are no other reports in the literature of Plg binding directly to the mesothelial cell surface via PlgRs, though mesothelial cells are known producers of Plg activators and inhibitors. Since Plg activation can drive various cellular activities, including migration, ECM reorganization, and fibrinolysis, the data here suggest a novel role for Plg in mediating mesothelial cell activities through direct binding and activation at the cell surface.

## 4. Materials and Methods

### 4.1. Reagents and Antibodies

All reagents were commercially purchased as follows: Glu-Plg (R&D Systems), Lys-Plg (ThermoFisher, Waltham, MA, USA), FastGro synthetic FBS (MP Biomedicals, Solon, OH, USA), bovine serum albumin (BSA, Millipore Sigma, Burlington, MA, USA), UK371804 (Cayman Chemical, Ann Arbor, MI, USA), and ε-aminocaproic acid (EACA, MP Biomedicals, Solon, OH, USA). Glu-Plg or Lys-Plg were used experimentally at a final concentration of 1 µM to mimic normal serum Plg concentrations (0.8 µM) [25]. Primary antibodies were purchased from BD Biosciences and used diluted in 3% BSA/PB as follows: mouse anti-Plg (1:1000), rabbit anti-H2B (1:1000), rabbit anti-ENO-1 (1:1000), rabbit anti-ANXA2 (1:1000), mouse anti-CK8 (1:100), or mouse anti-Plg/Pla (1:2500). Secondary antibodies were used at 1:1000 dilution and included goat-anti-mouse IgG-Alexa Fluor 488 (ThermoFisher) and goat-anti-rabbit IgG-FITC (BD Biosciences, San Jose, CA, USA). All other reagents were purchased from Fisher Scientific (Hampton, NH, USA), unless specified.

### 4.2. Cell Cultures

All cells were cultured at 37 °C with 5% CO_2_. Non-malignant, transformed human pleural mesothelial cells, MeT-5A (ATCC, Manassas, VA, USA), were grown and maintained in RPMI medium supplemented with FBS (5%), penicillin/streptomycin (1%), L-glutamine (1%), and other essential amino acids (1%).

Human monocytes, THP-1 cells (ATCC, Manassas, VA, USA), were grown and maintained in RPMI medium supplemented with FBS (10%), penicillin/streptomycin (1%), L-Glutamine (1%), 2-mercaptoethanol (20 mM), and other essential amino acids (1%). THP-1 cells were differentiated into macrophages in medium supplemented with 14 ng/mL of phorbol 12-myristate 13-acetate (PMA) overnight.

Primary human peritoneal mesothelial cells (Coriell Institute for Medical Research, Camden, NJ, USA) were grown and maintained in Medium 199 media (Sigma Aldrich, Rockville, MD, USA) supplemented with 15% FBS and 1% L-Glutamine.

All cells were maintained in FBS for growth but were serum-starved prior to experimentation.

### 4.3. Fluorescent Microscopy

To visualize Plg binding, MeT5A cells (1 × 10^4^) were adhered to positively charged microscopy slides (Fisher Scientific, Hampton, NH, USA), then serum-starved overnight. Cells were fixed for 20 min in 2% paraformaldehyde, washed with PBS-T, and incubated for 1 h with human Plg (1 µM) and mouse anti-human Plg. The slides were again washed and incubated with FITC-conjugated secondary antibody for 1 h, washed, and incubated in 300 nM DAPI solution for 2.5 min. Slides were then washed, dried, sealed with VECTASHIELD (Vectorlabs, Newark, CA, USA), and imaged at 40× on Olympus FV1000 Confocal Microscope.

### 4.4. Flow Cytometry

To measure Plg and PlgRs, cells were collected and aliquoted into groups of 1 × 10^6^ cells, then washed 2× with FACS buffer (500 mL PBS, 0.5 g NaN_3_, 0.1% BSA, pH 7.7). Cells were pelleted by centrifugation, then incubated for 30 min with primary antibody. Cells were then washed twice with FACS buffer and incubated with secondary antibody for 15–30 min, again washed with FACS buffer, resuspended in FACS analysis buffer (250 mL PBS, 5 g NaN_3_, 1% BSA, pH 7.8), and analyzed on a FACSCalibur Flow Cytometer (Becton Dickinson, San Jose, CA, USA).

To measure differentiation, cells were seeded on a 96-well plate and treated with Plg, Plg and aPlg, or TGF-beta (2 ng/mL) for 48 or 96 h in FBS-free media. Treated cells were collected, washed twice with 1× Perm/Wash (BD Biosciences, San Jose, CA, USA), pelleted by centrifugation, then stained with primary antibody for 30 min. Cells were stained with either FITC-conjugated anti-SMA or unconjugated rat anti-vimentin, as previously described [35]. Cells were then washed twice with Perm/Wash and resuspended in FACS analysis buffer or incubated with anti-rat PE-conjugated anti-rat secondary antibody for 30 min, washed with FACS buffer, and resuspended in FACS analysis buffer. All samples were analyzed on a FACSCalibur Flow Cytometer (Becton Dickinson, San Jose, CA, USA).

All samples were analyzed by gating on live cells and geographic mean fluorescent intensity (gMFI) measured against background fluorescent isotype control cells. To correct for differences in inter-day runs, gMFIs were converted to a resolution metric (R_D_), which was then used for statistical analyses.

### 4.5. Plasmin Activity Assays

Cells were seeded at 1 × 10^5^ cells per well on a 96-well plate and adhered overnight. Cells were serum-starved overnight and then incubated with 1 µM Glu- or Lys-Plg, as specified in figure legends; Plg concentration was determined experimentally after testing doses from 1–10 µM (data not shown), and the lowest dose was chosen to mimic normal serum Plg concentrations [36]. A plasmin activity assay was then run according to the manufacturer’s specification (Plasmin Activity Assay Kit, Abcam, Cambridge, UK). Briefly, after cell incubation with test samples for 1–12 h, as indicated in figure legends, 50 µL of plasmin substrate was added to each well. Substrate cleavage was measured by fluorescence detection at Ex/Em 360/450 nm in kinetic mode (read every 2.5 min for 22 min) on a Synergy HTX Multi-Mode Microplate Reader. To measure activity in the supernatant, experiments were set up as above, then supernatants collected and moved to clean assay wells following a 1 h incubation with Plg. Supernatants were replaced on cells with serum-free media. Pla substrate was then added to cells and supernatants separately, and activity assessed as above.

### 4.6. Plg Blocking Assays

To test the effect of various compounds on Plg binding and activity, plasmin activity assays or flow cytometry were performed as above with the following modifications. For FBS analyses, FBS or FastGro synthetic FBS was added at a final concentration of 10% and incubated for 10 min prior to Plg addition. For EACA analyses, Plg was incubated with EACA at 1.6 mM final concentration in PBS for 30 min prior to addition to serum-starved cells. The final EACA concentration was chosen based on preliminary trials using 0.5–1.6 mM, a range based on other studies [37]; 1.6 mM inhibited Plg binding over a 12 h period (data not shown). For uPA analyses, the uPA inhibitor UK37180 was added to cells at a final concentration of 10 µM and incubated for 10 min prior to Plg addition. For PlgR blocking, cells were exposed to PlgR antibodies singularly or in combination and incubated for 30 min prior to Plg addition.

### 4.7. Proliferation Assays

MeT5A cells were seeded at 10–15 ×10^3^ cells/well on a 96-well plate and adhered overnight. Cells were treated with Plg or Plg + EACA (1.6 mM) for 48 h. The treatments were given at 1:1000 dilution in FBS-free media. Trials were also performed using media supplemented with synthetic FBS (FastGro, MP Biomedicals, Solon, OH, USA) at a final concentration of 5% to mimic FBS concentration in culture media. Cell number was determined using a CyQuant assay, run according to the manufacturer’s specification (CyQUANT, NF Assay Kit, ThermoFisher, Waltham, MA, USA). Proliferation was measured by comparing fluorescence intensity (i.e., DNA content) of treated cells to that of untreated control cells. Fluorescence was detected at 485/530 nm on a Synergy HTX Multi-Mode Microplate Reader.

### 4.8. Migration Assays

To examine cell chemotaxis, Transwell inserts (Corning) containing 5 µm pores were used. Serum-starved MeT5A cells were then seeded (5 × 10^3^) in FBS-free medium in the upper chambers of the Transwell insert and medium supplemented with 1% FBS was added to the lower chamber as a chemotactic. MeT5A cells were either treated with recombinant human Plg (1:1000) prior to seeding or were left untreated. Plates were incubated at 37 °C for 18 h, and then non-migrating cells were removed from the upper chambers by gently wiping with a Q-tip. Cells that migrated to the lower side of the insert were fixed in 2% paraformaldehyde for 10 min, stained with crystal violet, and visualized on a light microscope. Each insert was visualized at five randomly selected locations, and the cells counted. The mean number of cells migrating through the inserts was calculated based on the surface area (mm^2^) of the insert.

To examine cell invasion through a collagen matrix, Transwell inserts were used as above but coated with gel of type I rat tail collagen (R&D Systems, Minneapolis, MN, USA) prior to cell seeding. To prepare the collagen gels, collagen stock (5 mg/mL) was activated by mixing with 2 N NaOH, according to the manufacturer’s instructions, then diluted in 10× PBS and diH20 to a final concentration of 1.7 mg/mL or 2.65 mg/mL and 50 µL added to the upper chamber of the Transwell insert. Plates were incubated at 37 °C for 1 h to allow collagen gel to polymerize. Cells were then treated with Plg (1:1000), Plg + UK37180, or left untreated, then seeded onto the collagen gel (5000 cells/insert). Invasion was assessed at 24 h, as described for the chemotaxis assays.

### 4.9. Statistical Analysis

One-way ANOVA and Dunnett’s or Tukey’s HSD post hoc tests were performed using R (4.0.3). Statistical significance was defined as *p* = 0.05.

## Data Availability

Not applicable.
